# EPID‐based *in vivo* dosimetry using Dosimetry Check™: Overview and clinical experience in a 5‐yr study including breast, lung, prostate, and head and neck cancer patients

**DOI:** 10.1002/acm2.12441

**Published:** 2018-12-07

**Authors:** William H. Nailon, Daniel Welsh, Kim McDonald, Donna Burns, Julie Forsyth, Gillian Cooke, Francisco Cutanda, Linda J. Carruthers, Duncan B. McLaren, Josep Puxeu Vaqué, Terence Kehoe, Sankar Andiappa

**Affiliations:** ^1^ Department of Oncology Physics Edinburgh Cancer Centre Western General Hospital Edinburgh UK; ^2^ School of Engineering The University of Edinburgh The King's Buildings Edinburgh UK; ^3^ Department of Clinical Oncology Edinburgh Cancer Centre Western General Hospital Edinburgh UK

**Keywords:** dose verification, EPID dosimetry, *in vivo* dosimetry, transit dosimetry

## Abstract

**Background:**

Independent verification of the dose delivered by complex radiotherapy can be performed by electronic portal imaging device (EPID) dosimetry. This paper presents 5‐yr EPID *in vivo* dosimetry (IVD) data obtained using the Dosimetry Check (DC) software on a large cohort including breast, lung, prostate, and head and neck (H&N) cancer patients.

**Material and Methods:**

The difference between *in vivo* dose measurements obtained by DC and point doses calculated by the Eclipse treatment planning system was obtained on 3795 radiotherapy patients treated with volumetric modulated arc therapy (VMAT) (*n* = 842) and three‐dimensional conformal radiotherapy (3DCRT) (*n* = 2953) at 6, 10, and 15 MV. In cases where the dose difference exceeded ±10% further inspection and additional phantom measurements were performed.

**Results:**

The mean and standard deviation (μ±σ) of the percentage difference in dose obtained by DC and calculated by Eclipse in VMAT was: 0.19±3.89% in brain, 1.54±4.87% in H&N, and 1.23±4.61% in prostate cancer. In 3DCRT, this was 1.79±3.51% in brain, −2.95±5.67% in breast, −1.43±4.38% in bladder, 1.66±4.77% in H&N, 2.60 ± 5.35% in lung and −3.62±4.00% in prostate cancer. A total of 153 plans exceeded the ±10% alert criteria, which included: 88 breast plans accounting for 7.9% of all breast treatments; 28 H&N plans accounting for 4.4% of all H&N treatments; and 12 prostate plans accounting for 3.5% of all prostate treatments. All deviations were found to be as a result of patient‐related anatomical deviations and not from procedural errors.

**Conclusions:**

This preliminary data shows that EPID‐based IVD with DC may not only be useful in detecting errors but has the potential to be used to establish site‐specific dose action levels. The approach is straightforward and has been implemented as a radiographer‐led service with no disruption to the patient and no impact on treatment time.

## 1 INTRODUCTION

It is recommended that all radiotherapy centers in the United Kingdom have a protocol for accurately measuring the dose delivered to a patient during a course of radiotherapy and comparing this to the planned dose.^1^, ^2^, ^3^, ^4^ This approach, commonly known as transit or IVD, has its origins in the 1980s and 1990s when radiographic and radiochromic films were used for this purpose. More recently thermoluminescent detectors (TLDs), semiconductor diodes, metal‐oxide field effect transistors (MOSFETs), and optically stimulated luminescence dosimeters (OSLDs) have been used for point dose measurements.^5^, ^6^, ^7^ However, there are inherent difficulties with each of these approaches, which have been comprehensively reviewed in several key publications.^8^, ^9^, ^10^, ^11^ Many of the limitations of these dosimeters can be overcome by the use of EPIDs, which although developed primarily for imaging, are now widely used as dosimeters and consequently for treatment verification.^11^, ^12^, ^13^, ^14^, ^15^ EPIDs, like the aforementioned dosimeters, can be used for either pretreatment verification without the patient present or, as discussed here, for IVD where the patient is present. The main challenge in using EPIDs as dosimeters is in the mapping between the EPID images into dose, with two techniques commonly used for this purpose. In the first a portal dose image is predicted from the treatment plan and the computerized tomography (CT) images used for planning, which is compared to the measured portal dose image. In the second, the measured portal dose image is combined with a back‐projection algorithm to calculate the dose in any given CT voxel and hence received by a patient.

The most widespread use of EPIDs as dosimeters has been in pretreatment verification.^16^, ^17^, ^18^ However, there are limitations associated with pretreatment verification for detecting certain errors such as those associated with patient anatomy.^19^ Furthermore patient‐specific pretreatment verification requires additional quality assurance (QA) procedures and linear accelerator time. Using EPID‐based IVD for patient‐specific QA overcomes these weaknesses and allows verification of the radiotherapy workflow using an independent technique. This was demonstrated in practice in a large‐scale study of 4337 patients verified using *in vivo* dosimetry between 2005 and 2009 at the Netherlands Cancer Institute. Of the 17 serious errors detected nine would not have been detected by standard pretreatment verification.^19^ This was endorsed further in a recent large‐scale review by Mijnheer et al. 20 reporting on 3‐yr experience of 3D EPID IVD. Clinically relevant deviations were detected in approximately 1 in 430 patient treatments. These changes would not have been detected by pretreatment verification as they were as a result of deviations from routine clinical procedure and anatomical changes. There is also compelling evidence for treatment adaptation using dosimetric information acquired by this approach. In a recent study of five patients 3D EPID dose was combined with cone‐beam CT (CBCT) imaging to detect atelectasis‐induced dose changes in lung cancer patients where plan adaptation would be beneficial.^21^

There are now commercial systems available for IVD including: Epiqa by EPIdos, Bratislava, Slovakia;^22^ EPIGray by DOSIsoft;^14^ Dosimetry Check (DC) by Math Resolutions LLC, Columbia, MD, USA^23^, ^24^, ^25^, ^26^ and some institutions have developed in‐house solutions for IVD.^27^, ^28^ Clinical experience from these systems is accumulating with a recent study from the United Kingdom on 58 patients clearly demonstrating the advantage of the commercial *in vivo* dosimetry system EPIgray for detecting anatomical changes in three of the 20 prostate cancer patients in the cohort. All EPID images used in the study were acquired on Elekta Synergy and Precise linear accelerators (Elekta, Stockholm, Sweden) equipped with iView a‐Si portal imagers (Perkin Elmer, Beaconsfield, UK).^29^ Here, we present complementary data obtained from our 5‐yr experience of using the DC EPID IVD QA software on Varian C‐Series and Truebeam linear accelerators (Varian Medical Systems, Inc., Palo Alto, CA, USA) equipped with Varian a‐Si 1000 EPIDs on a range of tumor sites including: breast; prostate; H&N lung and brain cancer.

## 2 MATERIAL AND METHODS

### 2.A Dosimetry Check — technical overview

This section presents a technical overview of the DC software including general commissioning and the steps involved in configuring it for use with a Varian aSi 1000 EPID device fitted to a Varian C‐series linear accelerator. This information is also valid for other EPID/linear accelerator configurations.

The DC software has two main technical elements. The first is that there is a mapping between the EPID fluence and the monitor units (MU) that would produce the same exposure at the center of a 10 × 10 cm^2^ field at the appropriate reference conditions. The output of this mapping is termed the relative monitor unit (RMU). The second is that the scatter within the EPID housing must be taken into account to allow this new unit of RMU to independently calculate the dose received by a patient. This is done by deconvolution of the EPID fluence with the point spread function (PSF) of the EPID, which produces the RMU in terms of in‐air fluence.

In practice the PSF has to take into account the dependence of the EPID on the input beam energy and the additional low‐energy scatter radiation reaching the EPID from the presence of a patient in the beam. In DC this is done at commissioning by calculating the PSF for a beam incident on and exiting water at regular intervals from 5 cm up to a maximum of 60 cm between the EPID and the radiation source.

#### 2.A.1 Relative monitor units

To obtain the absorbed dose (cGy) from the EPID, integrated EPID images are first mapped to RMU, which was defined by Renner as the number of MU that produces the same EPID pixel gray levels as a well‐controlled calibration condition.^24^ This is usually the 10 × 10 cm^2^ reference field that is used to define the output of a linear accelerator, typically as, “1.0 cGy/MU at 10 × 10 cm^2^ field size at 100 cm from the surface of water at 1.5 cm depth for 6 MV x rays^a^.” In the case of open square fields this may be thought of as the collimator scatter factor (S_C_) multiplied by the output (MU).

The first step in converting an integrated EPID image into RMU is to establish the relationship between the EPID signal at the central axis of a 10 × 10 cm^2^ field and the corresponding MU required to obtain this signal. To account for points not on the central axis, or off‐axis points, the in‐air off‐axis ratio (OAR) along the diagonals of a 40 × 40 cm^2^ field are measured to obtain the average OAR. Multiplying EPID pixels at a distance r cm from the central axis by this value restores the horns on a crossbeam profile, which arise as a result of using a flattening filter.

#### 2.A.2 *In vivo* dose evaluation

From this knowledge of the fluence at each pixel of the EPID, which is in RMU, and the beam geometry and patient CT it is possible to ray trace from the x‐ray source through the equivalent thickness of water that would produce this fluence. The same principle is applied when the planning CT is used in place of water and ray tracing is used to establish the dose at a point in the CT and hence the patient. The fluence map collected by the EPID image is the source of input for the DC dose calculation engine. This fluence map is used to parameterize the independent pencil beam dose calculation (PBC) algorithm that is used by DC. The dose calculated by DC is next compared to the dose matrix calculated by the treatment planning system. Quantitative evaluation of the difference between the planned and measured dose distribution is carried out in DC using either whole volume or partial volume gamma analysis or by a point dose comparison.^30^

The DC software platform has been used for IVD at the Edinburgh Cancer Centre since 2011 to monitor 3795 patients treated with VMAT and 3DCRT at beam energies 6, 10, and 15 MV. This included the cancer sites abdominal (*n* = 38); brain (*n* = 256); breast (*n* = 1215); genitourinary (*n* = 246); pelvic (*n* = 318); H&N (*n* = 636); lung (*n* = 664); prostate (*n* = 345); other (*n* = 77).

### 2.B Dosimetry Check — clinical implementation

**Figure 1:**
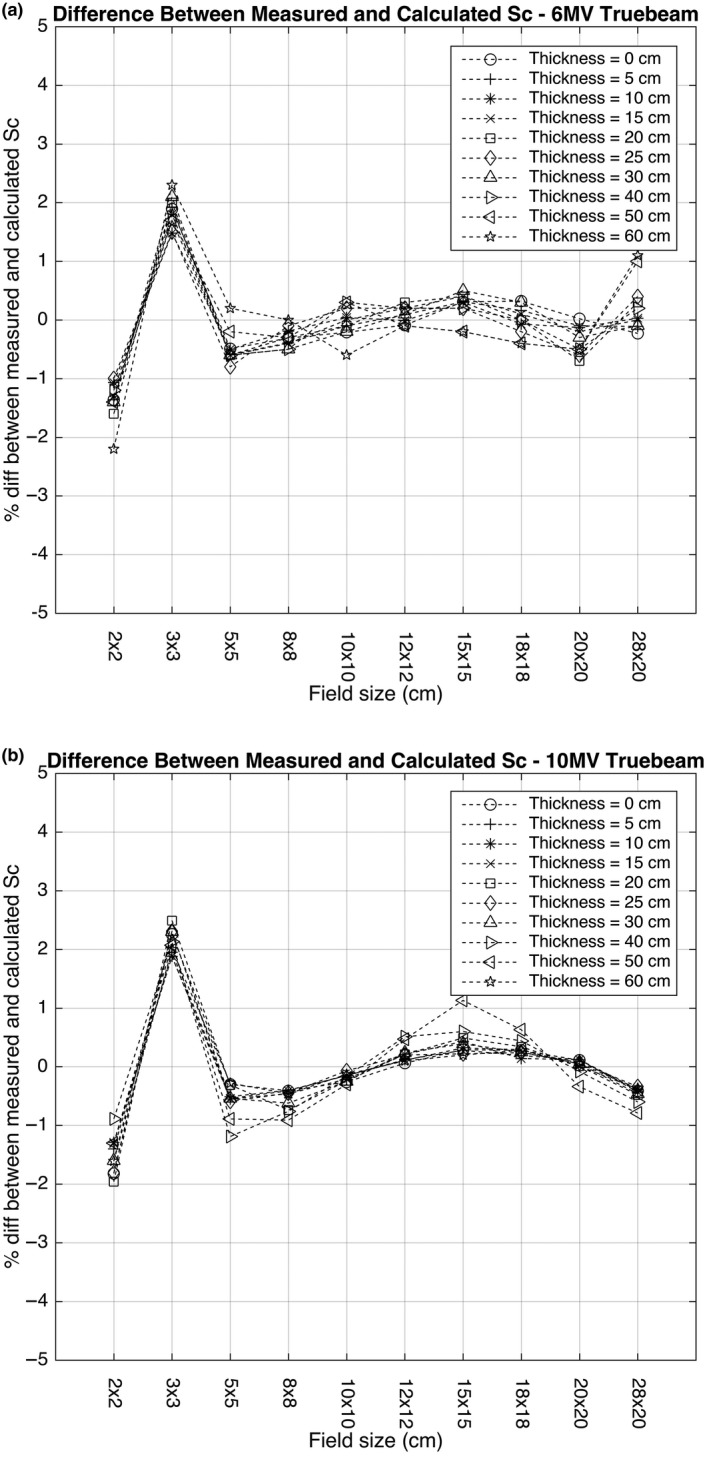
Percentage difference between $S_{C}^{\mathrm{MEAS}}$ to $S_{C}^{\mathrm{CALC}}$ on field sizes from 2 × 2 cm^2^ to 28 × 20cm^2^ at 10 water depths in the range 5 cm to 60 cm at a focus to imager distance (FID) of 150 cm at: (a) 6 and (b) 10 MV photon energies.

To configure the DC software for routine clinical use and to measure the dose received by a patient, EPID Sc measurements must be obtained at different beam energies at all available field sizes and at different water equivalent depths. Integrated EPID images of water were acquired on 10 field sizes ranging from 2 × 2 to 28 × 20 cm^2^ and 10 water depths in the range from 5 to 60 cm at a focus to imager distance (FID) of 150 cm and 100 MU. This procedure was repeated on the three C‐series and two Truebeam linear accelerators used at Edinburgh Cancer Centre. The FID was held constant and the treatment isocenter was always at the center of the water phantom. A fitting program, which is a standard module within DC, was used to fit the measured collimator scatter ($S_{C}^{\mathrm{MEAS}})$ to the calculated collimator scatter $\left(S_{C}^{\mathrm{CALC}}\right)$ using a downhill search optimization algorithm.^25^ Convergence was obtained when the variance, defined by,

(1) $$\sigma^{2}=\sum_{i}^{m}{\left(\frac{S_{C}^{\mathrm{MEAS}}-S_{C}^{\mathrm{CALC}}}{m}\right)}^{2}$$

over m data points was within 2%. Figure 1 shows the percentage difference between $S_{C}^{\mathrm{MEAS}}$ and $S_{C}^{\mathrm{CALC}}$ for 6 and 10 MV photon energies at a range of different field sizes. Following successful fitting of the data, it is possible to use DC to reconstruct the dose to a patient based on acquisition of an EPID integrated image.

Because DC is a completely independent verification system the treatment plan and associated CT images must be exported from the treatment planning software and manually imported into the DC software where reports are produced. Here, these were used to confirm the safe delivery of a treatment based on the Ionising Radiation (Medical Exposure) Regulations 2000 (IRMER)^31^ with alerts triggered when the dose difference between DC and Eclipse exceeded ±10% at the plan reference point. In the event that the dose exceeded this level, a full 3D gamma analysis was performed on the treatment plan with $\Delta D_{M}=4\%$ and $\Delta d_{M}=4\;\text{mm}$. This led to further positional and patient‐specific QA checks being performed.

## 3 RESULTS

### 3.A Conversion to RMU and data fitting

The EPID images acquired over the range of depths and field sizes previously defined were converted into RMU using an in‐built conversion function within DC. This function takes all of the DICOM images acquired at a particular depth as input and, together with an open field in‐air calibration image, performs the RMU conversion previously described.

**Figure 2:**
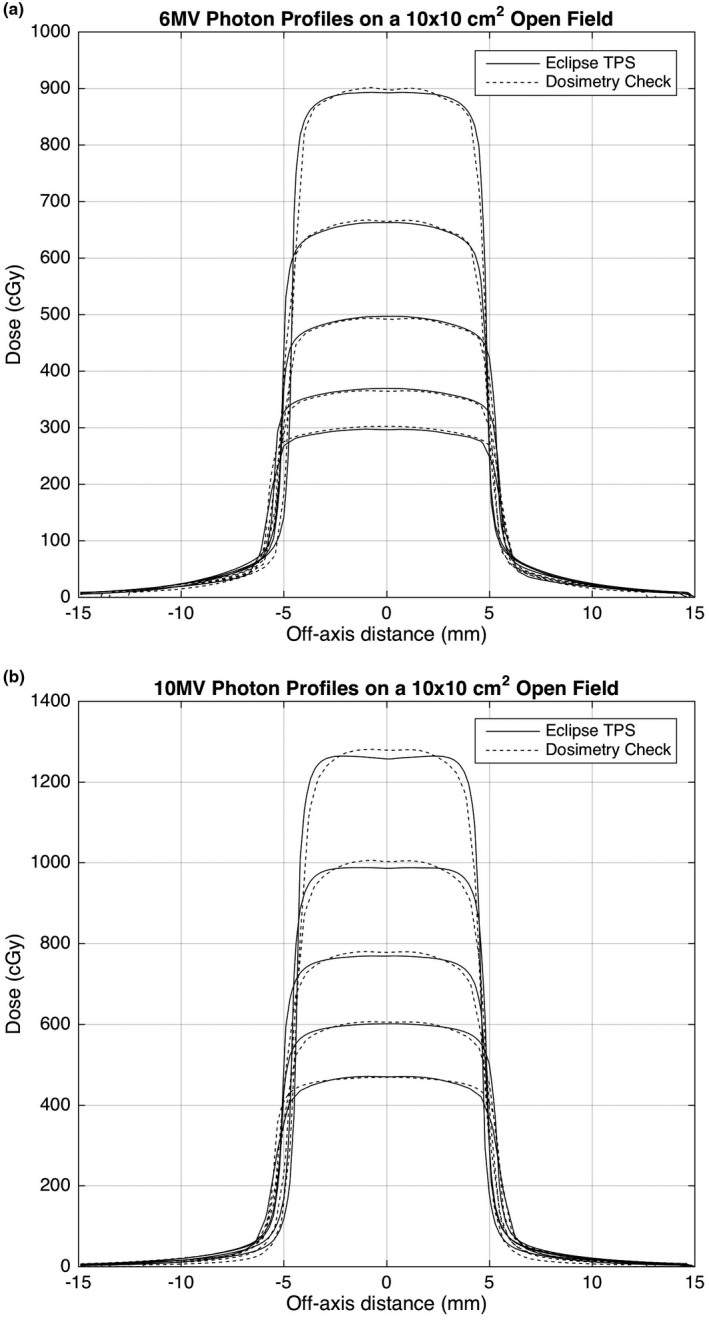
Profiles of measured dose and computed dose along the central axis of a 10 × 10 cm^2^ field irradiated by 6MV photons at depths of 1.5 cm, 5.0 cm, 10.0 cm, 15.0 cm, 20.0 cm, and 25 cm at (a) 6 and (b) 10 MV photon energies.

Fitting of the measured dose to water and the calculated dose to water by DC requires an initial estimate, which was first performed using the approach recommended by Math Resolutions. The percentage difference between $S_{C}^{\mathrm{MEAS}}$ and $S_{C}^{\mathrm{CALC}}$ at all depths was in general found to be within ±2.5% for all field sizes between 2 × 2 cm^2^ and 28 × 20 cm^2^. Figure 1 shows Sc on all fields and depths obtained on a Varian Truebeam linear accelerator with an aSI 1000 EPID on an Exact‐Arm at 6 and 10MV photon energies. Figure 2 shows the measured and computed dose profiles reconstructed from the EPID fluence along the central axis when a 6 and 10 MV photon beam was delivered to a 10 × 10 cm^2^ field at a range of depths. The computed dose profile was obtained using the Eclipse treatment planning system (Varian Medical Systems, Inc., Palo Alto, CA, USA).

### 3.B Radiotherapy courses and patients

**Figure 3:**
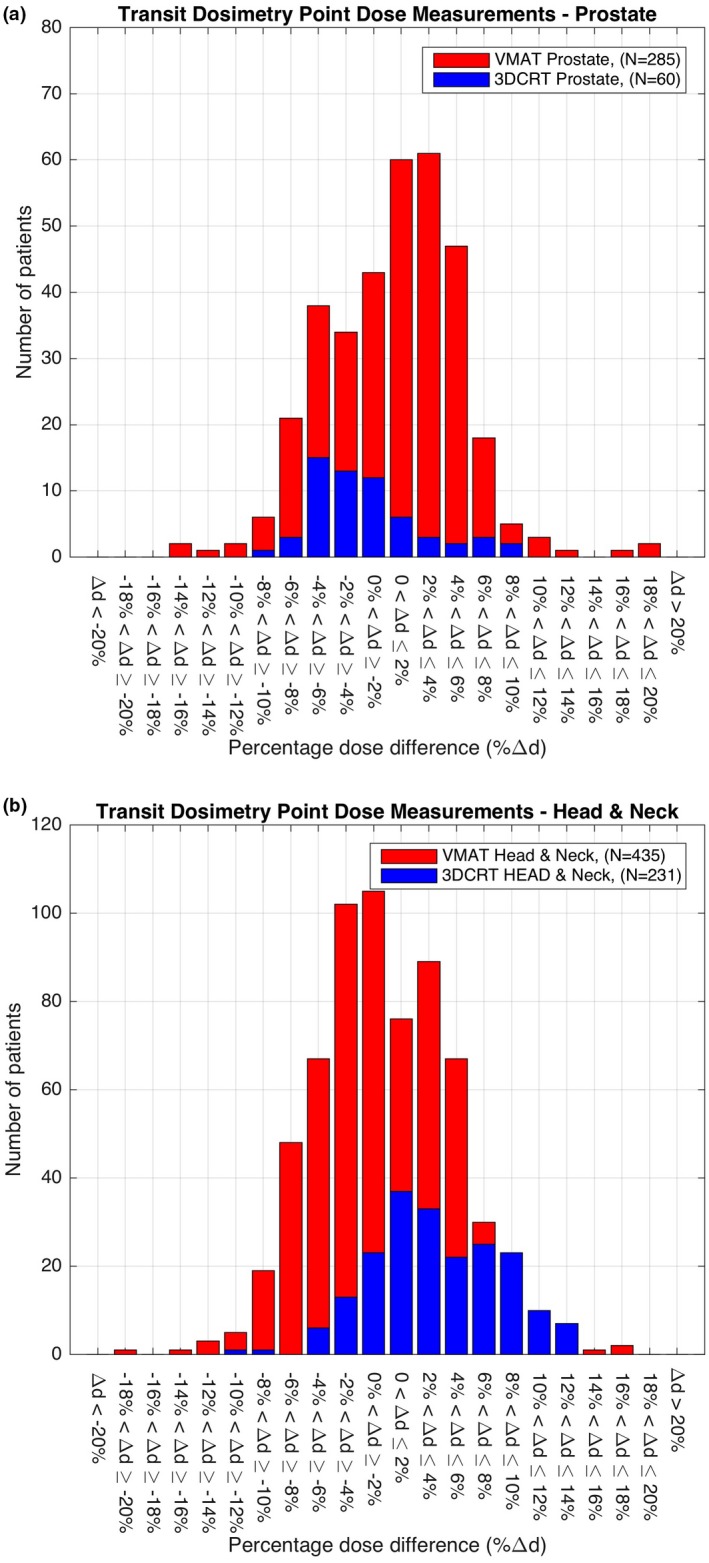
Transit dosimetry point dose measurements on: (a) prostate; and (b) H&N cancer patients treated between 2011 and 2016.

**Figure 4:**
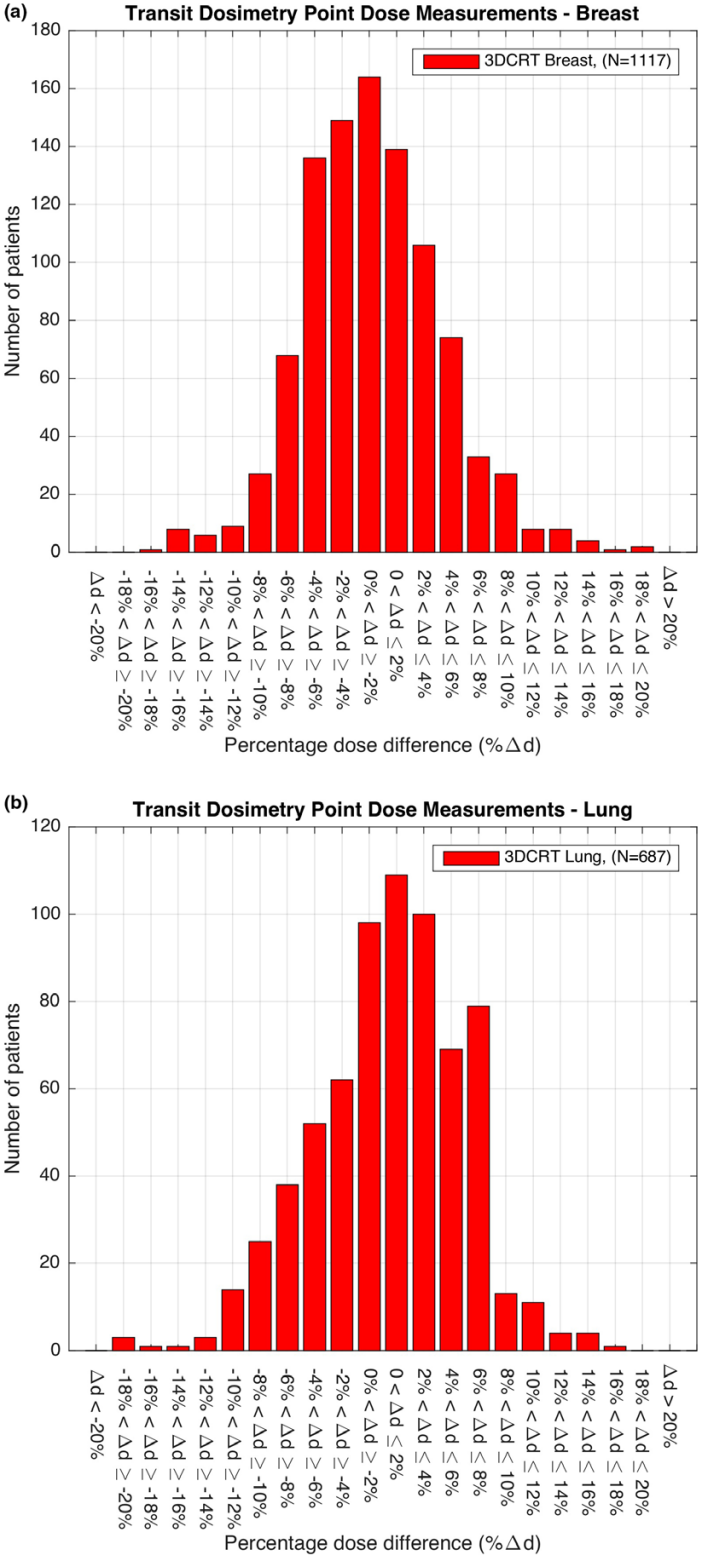
Transit dosimetry point dose measurements on: (a) breast; and (b) lung cancer patients treated between 2011 and 2016.

Figures 3 and 4 show the percentage difference in point doses calculated by Eclipse and measured by DC for all prostate, H&N, breast and lung cancer patients treated between 2011 and 2016. Table 1 shows the range of treatment sites, the total number of treatment plans verified and the alerts produced by the system. The mean and standard deviation (μ±σ) of VMAT cases was found to be 0.19 ± 3.89% in brain, 1.50 ± 4.87% in H&N, and 1.23 ± 4.61% in prostate cancer patients (Table 1). The mean and standard deviation of 3DCRT cases was found to be greater for each of these treatments, 1.79 ± 3.51% in brain, 1.66 ± 4.77% in H&N, and −3.62±4.00% in prostate cancer patients (Table 1). The total number of alerts produced at the ±10% action level used in routine clinical practice was 153 with the majority of the alerts found in breast cancer patients, *n* = 88, which represented 7.8% of all breast cancer patients monitored between 2011 and 2016. In the H&N cancer patient cohorts, there were 28 alerts representing 4.4% of the patients monitored and in prostate there were 12 alerts representing 3.5% of the patients monitored. In all of these cases further investigation using 3D gamma analysis and additional patient‐specific QA found no IRMER reportable treatment errors.

**Table 1:** Details of the range of treatment sites, total number of treatment plans verified and the alerts produced above the 10% threshold from 2011 to 2016

Treatment group/site	No. of Plans	Planning technique		Mean difference^a^ μ ± *σ*		No. of alerts
		VMAT	3DCRT	VMAT	3DCRT	>10%
Abdominal cancer	38					
Abdomen	30	4	26	–	1.75 ± 5.25	1
Pancreas	3	–	3	–	–	0
Spleen	2	–	2	–	–	0
Stomach	3	–	3	–	–	0
Brain cancer	256					
Brain	241	87	154	−0.19 ± 3.89	1.79 ± 3.51	2
Brainstem	5	2	3	–	–	0
Cavern sinus	1	1	–	–	–	0
Chordoma	1	1	–	–	–	0
Clivus	1	1	–	–	–	0
Meningioma	3	2	1	–	–	0
Pituitary	4	4	–	–	–	1
Breast cancer	1215					
Breast	1117	–	1117	–	−2.95 ± 5.67	88
Chest wall	91	–	91	–	0.30 ± 6.22	6
Lymphatics	7	–	7	–	–	1
Genitourinary cancer	246					
Anus	27	5	22	–	−4.99 ± 2.46	0
Esophagus	61	2	59	–	1.03 ± 4.97	3
Rectum	158	–	158	–	−2.77 ± 4.12	0
Pelvic cancer	318					
Bladder	104	4	100	–	−1.43 ± 4.38	2
Cervix	42	–	42	–	−4.49 ± 4.61	3
Endometrium	68	–	68	–	−5.58 ± 3.19	1
Gynecological	53	–	53	–	−3.74 ± 4.47	0
Pelvis	47	–	47	–	−3.76 ± 4.55	1
Uterus	1	–	1	–	–	0
Vagina	2	–	2	–	–	0
Vulva	1	–	1	–	–	0
Head and neck cancer	636					
Head and Neck	636	435	201	1.50 ± 4.87	1.66 ± 4.77	28
Lung cancer	664					
Lung	663	1	662	–	2.61 ± 5.35	2
Alveleolus	1	1	–	–	–	0
Prostate cancer	345					
Prostate	345	285	60	1.23 ± 4.61	−3.62 ± 4.00	12
Other cancers	77					
Miscellaneous	77	7	70			2
Total	3795	842	2953	–	–	153

a Mean and standard deviation are provided only where there are sufficient statistical data.

## 4 DISCUSSION

With the significant advances in radiotherapy delivery techniques such as IMRT and VMAT and their widespread adoption there is a pressing need for improved QA procedures that check the validity of these techniques in a clinically acceptable timeframe. Moreover it is now recommended, “for most patients^b^”, that there is an independent IVD verification of the dose delivered from complex radiotherapy treatments at the beginning of treatment.^1^ Many departments have investigated the use of different detectors such as TLDs, semiconductor diodes, and MOSFETS for IVD, however, the efficacy of these detectors is significantly affected by positioning, dose gradient and the increased QA time required to acquire and process the readings. The results presented here demonstrate that EPID‐based IVD using DC is a practical method for monitoring patients during treatment.

What is interesting to note from the 3DCRT IVD results is the diversity in the percentage dose difference between DC and Eclipse in breast, lung, H&N, and prostate cancer patients. In breast there was a ‐2.95% mean difference between the dose calculated in Eclipse and the IVD dose at a defined reference point. In lung there was a 2.6% difference, in H&N a 1.66% difference and in prostate a −3.62% difference. While these results indicate that the IVD values obtained by measurement are, in general, within the departmental tolerance of ±10% for all treatment sites they highlight systematic differences in the point dose measurements used for verification of breast, lung, H&N, and prostate cancer treatments. This may be as a result of the different approaches used for planning these treatments and selection of the reference point. It may also be due to the fundamental technical differences between DC and Eclipse, which requires further investigation.

Ninety‐six percent of the 3795 patients included passed the departmental alert criteria set for an acceptable difference (±10%) between the planned and delivered (*in vivo*) dose. Of those cases that exceeded the ±10% tolerance the majority were found to be in patients with breast, prostate, and H&N cancer. The reason for the alerts in the breast group was due to several compounding factors. These were (a) changes in the volume of breast irradiated at each fraction due to the inherent difficulties in positioning of the breast; (b) chest wall irradiation, particularly the impact of rib structures in the field and the resulting uncertainties in the dose; (c) in nodal breast irradiation where the EPID imager position has to be shifted, no off‐axis correction is currently applied in the calculation; (d) currently breast patients are treated in free‐breathing mode, adopting a breath‐hold technique will improve positioning and reduce dosimetric uncertainty.

In the prostate cohort the failures were due to bladder and rectal filling and in the H&N cancer group the alerts were as a result of weight loss and choice of the reference point used for analysis.

In the future the availability of IVD data, such as the data presented here, could be a powerful indicator of suboptimal treatments if correlated with long‐term patient follow‐up or outcome data. It could also be used, if processed immediately, to identify the correctness of individual multileaf collimator (MLC) fields 32 and to identify significant anatomical changes such as those seen in H&N cancer patients experiencing weight loss during treatment.^33^

After the DC software obtains the in‐air fluence in RMU it is possible to independently calculate the dose delivered to a given patient using the original planning CT scan. Currently the DC software uses a PBC algorithm to calculate this dose while the Eclipse treatment planning system used the Analytical Anisotropic Algorithm (AAA).^34^ The AAA algorithm has been shown to result in a lower mean dose than the PBC and in general a reduction in dose to the planning target volume (PTV), which may in part account for the differences between the calculated and measured doses.^35^ Other factors that contribute to the difference in dose observed in Fig. 2 include the EPID detector off‐axis energy response and the fitting parameters for all field sizes and depths acquired by DC. To account for a portion of these differences a collapsed cone algorithm is currently under development by Math Resolutions, which will improve the accuracy of the dose calculation in DC and ultimately reduce the variability in the results.^36^

The percentage difference between the measured and calculated Sc was found to be within ±1.0% for field sizes between 5 × 5 cm^2^ and 20 × 20 cm^2^. The largest difference was found at the smallest (2 × 2 cm^2^ and 3 × 3 m^2^) and largest (28 × 20 cm^2^) field sizes investigated. With the substantial increase in the use of small radiotherapy fields, particularly for hypofractionation techniques where high doses per fraction are delivered, it is important to obtain a better match of Sc.^37^

The role of checking has been identified as a key element when independently verifying the integrity of a treatment plan.^38^ The Edinburgh Cancer Centre has implemented a radiographer‐led IVD service for the vast majority of patients treated with radical intent. This has not only improved the efficiency and widespread deployment of this service but more importantly by involving radiographers at this stage of the radiotherapy chain a further check, independent of the existing MU check, has been introduced.

**Figure 5:**
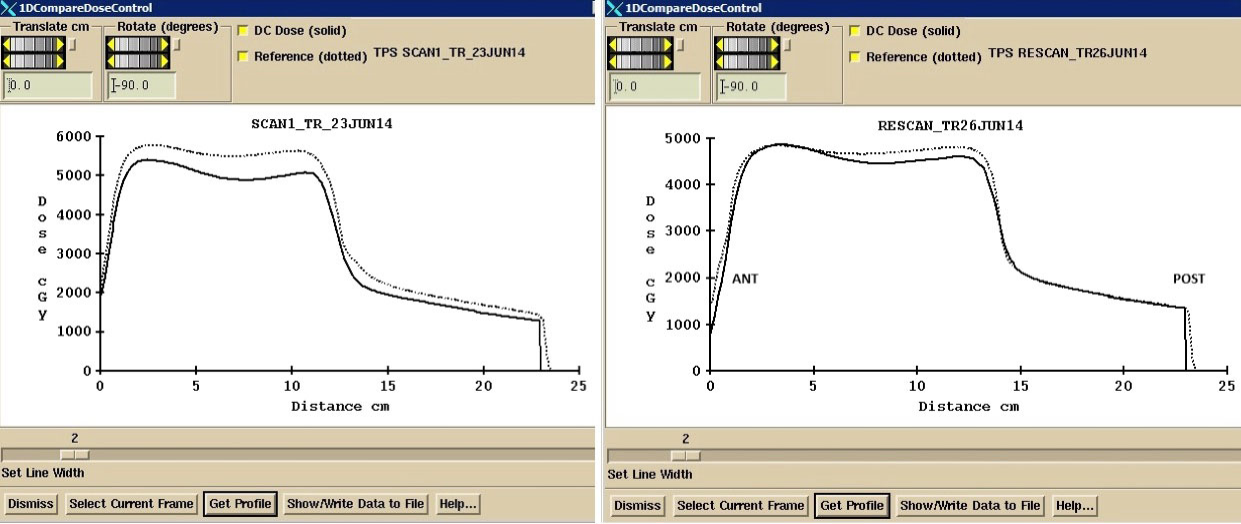
One‐dimensional dose profile through the bladder reference point in the AP direction. Left: Profile through the original CT scan. Right: Profile through the rescanned CT after further investigation with IVD.

Presently the DC software is used for all radical treatments at the Edinburgh Cancer Centre where the field dimensions do not exceed the EPID imager dimensions. Since full clinical implementation DC‐based IVD has not identified any reportable incidents and has proven to be invaluable at identifying and correcting for anatomical changes such as in the case where the difference between DC IVD and Eclipse was −10.65% at the reference point of a bladder cancer patient. A decision was taken to repeat DC IVD at the second fraction where the difference was −11.80%, which prompted a closer inspection of the treatment plan. This revealed that there was an excessive amount of bowel gas at the time of the treatment planning CT, which was not present at the time of treatment. The patient was rescanned, replanned, treated, and DC IVD used to confirm that the changes made were indeed appropriate. Figure 5 shows the 1D profile through the bladder in the anterior posterior (AP) direction in which it is clear that there is a much better match between the measured and planned dose after the rescan. The use of DC IVD for this case proved particularly helpful.

## 5 CONCLUSIONS

The ability to perform patient‐specific QA is now an accepted requirement in modern radiotherapy. This paper presents preliminary data, with a focus on safety, showing that EPID‐based IVD with DC has significant potential for this. From knowledge of the expected difference between the *in vivo* dose and the planned dose, collected on a large number of cases, it may be possible to set site‐specific alert criteria for a given treatment site. Furthermore, this approach has the potential to identify suboptimal treatments much earlier than is currently possible.

## CONFLICT OF INTEREST

The authors report a continuing nonfinancial collaboration with Math Resolutions. The authors report no other conflicts of interest. The authors alone are responsible for the content and writing of the paper.

## NOTES

^a^This is the definition of machine output at the Edinburgh Cancer Centre.

^b^This phrase was extracted verbatim from Ref. 1

## References

[1] The Royal College of Radiologists, Society and College of Radiographers, Institute of Physics and Engineering in Medicine, National Patient Safety Agency, British Institute of Radiology Towards Safer Radiotherapy. RCR; 2008.

[2] Alber M , Broggi S , De Wagter C , et al. Guidelines for the verification of IMRT. ESTRO Booklet No. 9. In: Mijnheer B, Georg D, eds. 1st ed Brussels (Belgium): ESTRO; 2008.

[3] Ezzell GA , Galvin JM , Low D , et al. Guidance document on delivery, treatment planning, and clinical implementation of IMRT: report of the IMRT subcommittee of the AAPM radiation therapy committee. Med Phys. 2003;30:2089e115. 12945975 10.1118/1.1591194

[4] Hartford AC , Palisca MG , Eichler TJ , et al. American Society for Therapeutic Radiology and Oncology (ASTRO) and American College of Radiology (ACR) practice guidelines for intensity modulated radiation therapy (IMRT). Int J Radiat Oncol Biol Phys. 2009;73:9e14. 19100920 10.1016/j.ijrobp.2008.04.049

[5] Essers M , Hoogervosrt BR , van Herk M , Lanson H , Mijnheer BJ . Dosimetric characteristics of a liquid‐filled electronic portal imaging device. Int J Rad Oncol Biol Phys. 1995;33:1265–1272. 10.1016/0360-3016(95)00108-5 7493851

[6] American Association of Physicists in Medicine (AAPM) . Diode in vivo dosimetry for patients receiving external beam radiation therapy. AAPM Report 87; 2005.

[7] Van Dam J , Marinello G . Methods for in vivo dosimetry in external radiotherapy. ISBN 90‐804532‐9, ESTRO 2006, Mounierlaan 83/12 – 1200 Brussels (Belgium); 2006.

[8] Edwards CR , Mountford PJ . Characteristics of in vivo dosimetry. BJR. 2009;82:3. 10.1259/bjr/32563777 19752169

[9] Mijnheer B . State of the art of in vivo dosimetry. Radiat Prot Dosimetry. 2008;131:117–122. 18765403 10.1093/rpd/ncn231

[10] Essers M , Mijnheer B . In vivo dosimetry during external photon beam radiotherapy. Int J Radiat Oncol Biol Phys. 1999;43:245–259. 10030247 10.1016/s0360-3016(98)00341-1

[11] Antonuk LE . Electronic portal imaging devices: a review and historical perspective of contemporary technologies and research. Phys Med Biol. 2002;47:R31–R65. 11936185

[12] Mijnheer B , Beddar S , Izewska J , Reft C . In vivo dosimetry in external beam radiotherapy. Med Phys. 2013;40:1–19. 10.1118/1.4811216 23822404

[13] Rozendaal RA , Mijnheer BJ , van Herk M , Mans A . In vivo portal dosimetry for head‐and‐neck VMAT and lung IMRT: linking *γ*‐analysis with differences in dose‐volume histograms of the PTV. Radiother Oncol. 2014;112:396–401. 24861630 10.1016/j.radonc.2014.03.021

[14] Francois P , Boissard P , Berger L , Mazal A . In vivo dose verification from back projection of a transit dose measurement on the central axis of photon beams. Phys Med. 2011;27:1–10. 20615735 10.1016/j.ejmp.2010.06.002

[15] Fidanzio A , Cilla S , Greco F , et al. Generalized EPID calibration for in vivo transit dosimetry. Phys Med. 2011;27:30–38. 20199885 10.1016/j.ejmp.2010.02.002

[16] Sharma DS , Mhatre V , Heigrujam M , Talapatra K , Mallik S . Portal dosimetry for pretreatment verification of IMRT plan: a comparison with 2D ion chamber array. J Appl Clin Med Phys. 2010;11:238–248. 10.1120/jacmp.v11i4.3268 PMC5720403 21081884

[17] Berry L , Sheu RD , Polvorosa CS , Wuu CS . Implementation of EPID transit dosimetry based on through‐air dosimetry algorithm. Med Phys. 2012;39:87–98. 22225278 10.1118/1.3665249

[18] Bojechko C , Phillps M , Kalet M , Forda EC . A quantification of the effectiveness of EPID dosimetry and software‐based plan verification systems in detecting incidents in radiotherapy. Med Phys. 2015;42:5363–5369. 26328985 10.1118/1.4928601

[19] Mans A , Wendling M , McDermott LN , et al. Catching errors with *in vivo* EPID dosimetry. Med Phys. 2010;37:2638–2644. 20632575 10.1118/1.3397807

[20] Mijnheer BJ , Gonzalez P , Olaciregui‐Ruiz I , Rozendaal RA , van Herk M , Mans A . Overview of 3‐year experience with large‐scale electronic portal imaging device‐based 3‐dimensional transit dosimetry. Pract Rad Oncol. 2015;5:679–687. 10.1016/j.prro.2015.07.001 26421834

[21] Persoon LCGC , Egelmeer AGTM , Ollers MC , Nijsten SMJJG , Troost EGC , Verhaegen F . First clinical results of adaptive radiotherapy based on 3D portal dosimetry for lung cancer patients with atelectasis treated with volumetric modulated arc therapy (VMAT). Acta Oncol. 2013;52;1484–1489. 24000957 10.3109/0284186X.2013.813642

[22] Epiqa in clinical practice. [cited 2016 June 13]. Available from http://www.wienkav.at/kav/kfj/91033454/physik/pd/epiqa.htm

[23] Pinkerton A , Hannon M , Kwag J , Renner WD . Experience using dosimetry check software for IMRT and Rapid‐Arc patient pre‐treatment QA and a new feature for QA during treatment. IC3DDose. J Phys: Conf Ser 2010;250:012101.

[24] Renner WD , Mehrdad S , Earl MA , Yu CX . A dose delivery verification method for conventional and intensity‐modulated radiation therapy using measured field fluence distributions. Med Phys. 2003;30:2996–3005. 14655947 10.1118/1.1610771

[25] Renner WD , Norton K , Holmes T . A method for deconvolution of integrated electronic portal images to obtain incident fluence for dose reconstruction. J App Clin Med Phys. 2005;6:22–39. 10.1120/jacmp.v6i4.2104 PMC5723452 16421498

[26] Renner WD . 3D dose reconstruction to insure correct external beam treatment of patients. Med Dosim. 2007;32:157–165. 17707194 10.1016/j.meddos.2007.02.005

[27] Piermattei A , Greco F , Azario L , et al. A National project for in vivo dosimetry procedures in radiotherapy: first results. Nucl Instrum Methods Phys Res B. 2012;B274:42–50.

[28] Wendling M , McDermott LN , Mans A , Sonke JJ , Van Herk M , Mijnheer BJ . A simple back projection algorithm for 3D in vivo EPID dosimetry of IMRT treatments. Med Phys. 2009;36:3310–3321. 19673227 10.1118/1.3148482

[29] Ricketts K , Navarro C , Lane K , et al. Clinical experience and evaluation of patient treatment verification with a transit dosimeter. Int J Radiat Oncol Biol Phys. 2016;95:1513–1519. 27262359 10.1016/j.ijrobp.2016.03.021

[30] Low DA , William BH , Mutic S , Purdy JA . A technique for the quantitative evaluation of dose distributions. Med Phys. 1998;25:656–661. 9608475 10.1118/1.598248

[31] Statutory Instrument 2000 No. 1059, The ionising radiations (Medical Exposures) Regulations. London: HMSO; 2000 http://www.opsi.gov.uk/si/si2000/20001059.htm

[32] Van Esch A , Depuydt T , Huyskens DP . The use of aSi‐based EID for routine absolute dosimetric pre‐treatment verification of dynamic IMRT fields. Radiother Oncol. 2004;71:223–234. 15110457 10.1016/j.radonc.2004.02.018

[33] Rozendaal RA , Mijnheer BJ , Hamming‐Vrieze O , Man A , van Herk M . Impact of daily anatomical changes on EPID‐based in vivo dosimetry of VMAT treatments of head‐and‐neck cancer. Radiother Oncol. 2015;116:70–74. 26142267 10.1016/j.radonc.2015.05.020

[34] Fogliata A , Nicolini G , Vanetti E , Clivio A , Cozza L . Dosimetric validation of the anisotropic analytical algorithm for photon dose calculation: fundamental characterization in water. Phys Med Biol. 2006;51:1421–1438. 16510953 10.1088/0031-9155/51/6/004

[35] Karlsson A , Behrens C , Ottosson R , Samsoe E , Sjostrom D . Investigation of the difference in PB and AAA calculated dose distributions for dynamic IMRT. Radiother Oncol. 2009;92:S193.

[36] Ahnesjö A . Collapsed cone convolution of radiant energy for photon dose calculation in heterogenous media. Med Phys. 1989;16:577–592. 2770632 10.1118/1.596360

[37] Zhu TC , Ahnesjö A , Lam KL , et al. Report of AAPM Therapy Physics Committee Task Group 74: in‐air output ratio, *S*c, for megavoltage photon beams. Med Phys. 2009;36:5261–5291. 19994536 10.1118/1.3227367

[38] Wiliams MV . Radiotherapy near misses, incidents and errors: radiotherapy incident in Glasgow. Clin Oncol. 2007;19:1–3. 10.1016/j.clon.2006.12.004 17305249

